# Quantified health and cost effects of faster endovascular treatment for large vessel ischemic stroke patients in the Netherlands

**DOI:** 10.1136/neurintsurg-2020-017017

**Published:** 2021-01-21

**Authors:** Henk van Voorst, Wolfgang G Kunz, Lucie A van den Berg, Manon Kappelhof, Floor M E Pinckaers, Mayank Goyal, Myriam G M Hunink, Bart J Emmer, Maxim J H L Mulder, Diederik W J Dippel, Jonathan M Coutinho, Henk A Marquering, Hieronymus D Boogaarts, Aad van der Lugt, Wim H van Zwam, Yvo B W E M Roos, Erik Buskens, Marcel G W Dijkgraaf, Charles B L M Majoie, For the MR CLEAN Registry investigators, Diederik W J Dippel

**Affiliations:** 1 Department of Radiology and Nuclear Medicine, Amsterdam University Medical Centers, location AMC, University of Amsterdam, Amsterdam, The Netherlands; 2 Department of Biomedical Engineering and Physics, Amsterdam University Medical Centers, location AMC, University of Amsterdam, Amsterdam, The Netherlands; 3 Department of Radiology, University Hospital, LMU Munich, Münich, Germany; 4 Department of Neurology, Amsterdam University Medical Centers, location AMC, University of Amsterdam, Amsterdam, The Netherlands; 5 Department of Radiology and Nuclear Medicine, Maastricht University Medical Center, Maastricht, The Netherlands; 6 Department of Radiology and Clinical Neurosciences, University of Calgary, Calgary, Alberta, Canada; 7 Department of Clinical Epidemiology, Erasmus University Medical Center, Rotterdam, The Netherlands; 8 Departments of Radiology, Erasmus MC University Medical Center, Rotterdam, The Netherlands; 9 Department of Neurology, Erasmus University Medical Center, Rotterdam, The Netherlands; 10 Department of Neurosurgery, Radboud University Medical Center, Nijmegen, The Netherlands; 11 Department of Epidemiology, University Medical Centre Groningen, Groningen, The Netherlands; 12 Department of Epidemiology and Data Science, Amsterdam University Medical Centers, location AMC, University of Amsterdam, Amsterdam, The Netherlands

**Keywords:** economics, stroke, thrombectomy, intervention, artery

## Abstract

**Background:**

The effectiveness of endovascular treatment (EVT) for large vessel occlusion (LVO) stroke severely depends on time to treatment. However, it remains unclear what the value of faster treatment is in the years after index stroke. The aim of this study was to quantify the value of faster EVT in terms of health and healthcare costs for the Dutch LVO stroke population.

**Methods:**

A Markov model was used to simulate 5-year follow-up functional outcome, measured with the modified Rankin Scale (mRS), of 69-year-old LVO patients. Post-treatment mRS was extracted from the MR CLEAN Registry (n=2892): costs per unit of time and Quality-Adjusted Life Years (QALYs) per mRS sub-score were retrieved from follow-up data of the MR CLEAN trial (n=500). Net Monetary Benefit (NMB) at a willingness to pay of €80 000 per QALY was reported as primary outcome, and secondary outcome measures were days of disability-free life gained and costs.

**Results:**

EVT administered 1 min faster resulted in a median NMB of €309 (IQR: 226;389), 1.3 days of additional disability-free life (IQR: 1.0;1.6), while cumulative costs remained largely unchanged (median: -€15, IQR: −65;33) over a 5-year follow-up period. As costs over the follow-up period remained stable while QALYs decreased with longer time to treatment, which this results in a near-linear decrease of NMB. Since patients with faster EVT lived longer, they incurred more healthcare costs.

**Conclusion:**

One-minute faster EVT increases QALYs while cumulative costs remain largely unaffected. Therefore, faster EVT provides better value of care at no extra healthcare costs.

## Introduction

Occlusions of the intracranial carotid artery and middle cerebral artery, commonly referred to as large vessel occlusions (LVO), are severe causes of acute ischemic stroke (AIS), frequently resulting in persisting neurological deficits affecting patients’ health and healthcare costs.^1–3^ Mortality of the LVO stroke population is high and a large portion of the survivors remain dependent on care.^2 3^ These outcomes have drastically improved in recent years with the introduction of endovascular treatment (EVT).^4^ The beneficial effect of EVT is, however, highly time-dependent.^5^ As a result, various policies that aim at reducing time delay from onset of neurologic deficit to EVT have been investigated.^6^ However, it remains unclear what the health and cost effects of faster EVT are in the years following treatment.

Health outcomes and costs have a strong association in the case of LVO stroke as the high disease burden results in a lower perceived quality of life and higher demand on healthcare facilities.^3 7^ Besides the short- and long-term health benefits, several studies have proven that EVT reduces healthcare costs in the years after treatment.^4 8 9^ Although EVT has become standard practice in developed countries worldwide,^10^ various time-consuming inefficiencies remain present in current practice.^11 12^ With the growing use of advanced diagnostics the negative side-effects in terms of added delay to treatment should be known.^13^ With several policies aiming at faster EVT delivery, proposed and promising yet time-consuming advanced diagnostics available,^6 13^ so understanding the value of each minute has become increasingly important.

Studies that evaluate workflow improvements mainly used time saved as primary outcome and thus do not include long-term health and cost effects.^6^ A recent study determined the health and cost effects of faster EVT in the case of LVO stroke based on US guidelines and data.^14^ However, extrapolation of US data on a population level to the Netherlands and other European countries is difficult: besides differences in healthcare costs, geographical differences cause different onset to EVT times,^15 16^ and US guidelines apply stricter imaging selection criteria for EVT eligibility.^17 18^ The use of similar guidelines and empirical findings suggest that Dutch data is more comparable to other European countries.^19^ Furthermore, the adaptation of the guidelines in the US is not universal, resulting in a more comparable situation in regions of the US to the Dutch setting. The aim of this study was to quantify the value of faster EVT in terms of health and healthcare costs for the Dutch LVO stroke population with a Markov model.

## Methods

In this study, simulations with a Markov model were performed to compute expected health and costs during follow-up for each hour of delay to EVT of patients with intracranial carotid artery, M1, and proximal M2 occlusions. Subsequently, the differences in health and cost outcomes between each hour of delay was used to represent the effect of faster EVT per minute.

### Modeling procedure

**Figure 1:**
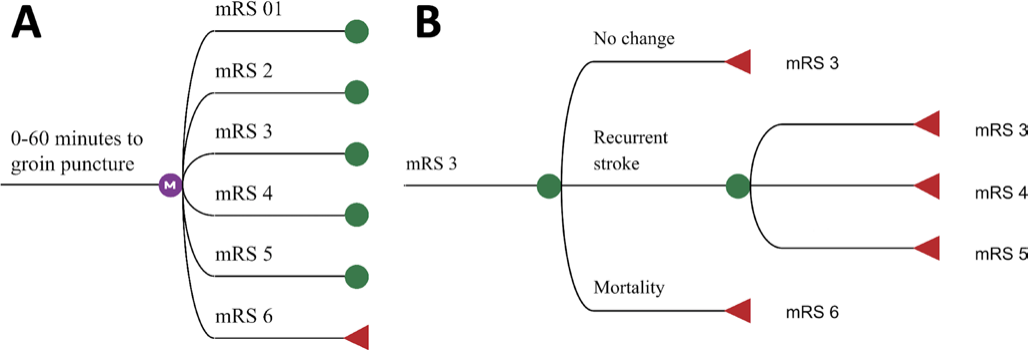
Markov model architecture: Pane A: Short-term model used for each hour to simulate 90-day mRS (modified Rankin Scale). In this example, 0–60 minutes of onset time to groin puncture was presented. Pane B: Long-term model for patients (example for mRS 3 at 90-days' post-index stroke). After recurrent stroke an equal or higher mRS score can be achieved. After stroke recurrence, death (mRS 6) is not possible to prevent duplicate mortality rates in the model. mRS after stroke recurrence was based on normalized values from the MR CLEAN trial control arm.

A two-staged Markov model was developed with a short-term and long-term part. For each time step, simulated patients could be in one of six Markov states defined by modified Rankin Scale (mRS) sub-scores: mRS 0 and 1 were combined in one Markov state since the sample size was otherwise too low to retrieve accurate estimates. The mRS is a seven-point scale for disability, ranging from 0 (no disabilities) to 6 (death). The short-term model was used to simulate 90-day mRS per hour delay of time from onset to the start of EVT, which was defined by the time of groin puncture. Time to groin puncture was preferred to time to end-of-intervention as the resulting time related mRS would include an effect related to LVO and thus intervention complexity: furthermore it is unlikely that intervention time can be saved to achieve faster EVT. Simulated cohorts were assumed to exist of an even split of males and females aged 69, the median age of patients in the MR CLEAN registry,^15^ at the beginning of the long-term simulations deterioration of mRS could only occur after stroke recurrence or due to death. In the long-term model changes in mRS due to stroke recurrence and all-cause mortality were simulated over 5 years of follow-up with a cycle length of 1 year. Figure 1 contains the short- and long-term model architecture graphically. Quality-adjusted life years (QALYs) and costs related to each Markov state were discounted at a 1.5% and 4% compounded annual rate, respectively, according to Dutch guidelines for cost effectiveness research.^20^ A yearly inflation rate of 1.7% was used to adjust future costs based on forecasts from the Dutch Ministry of Health, Welfare and Sport.^21^ TreeAge software (TreeAge Pro 2019, version R2.1; TreeAge, Williamstown MA, USA) was used for implementing the model and simulations.

### Data sources

**Figure 2:**
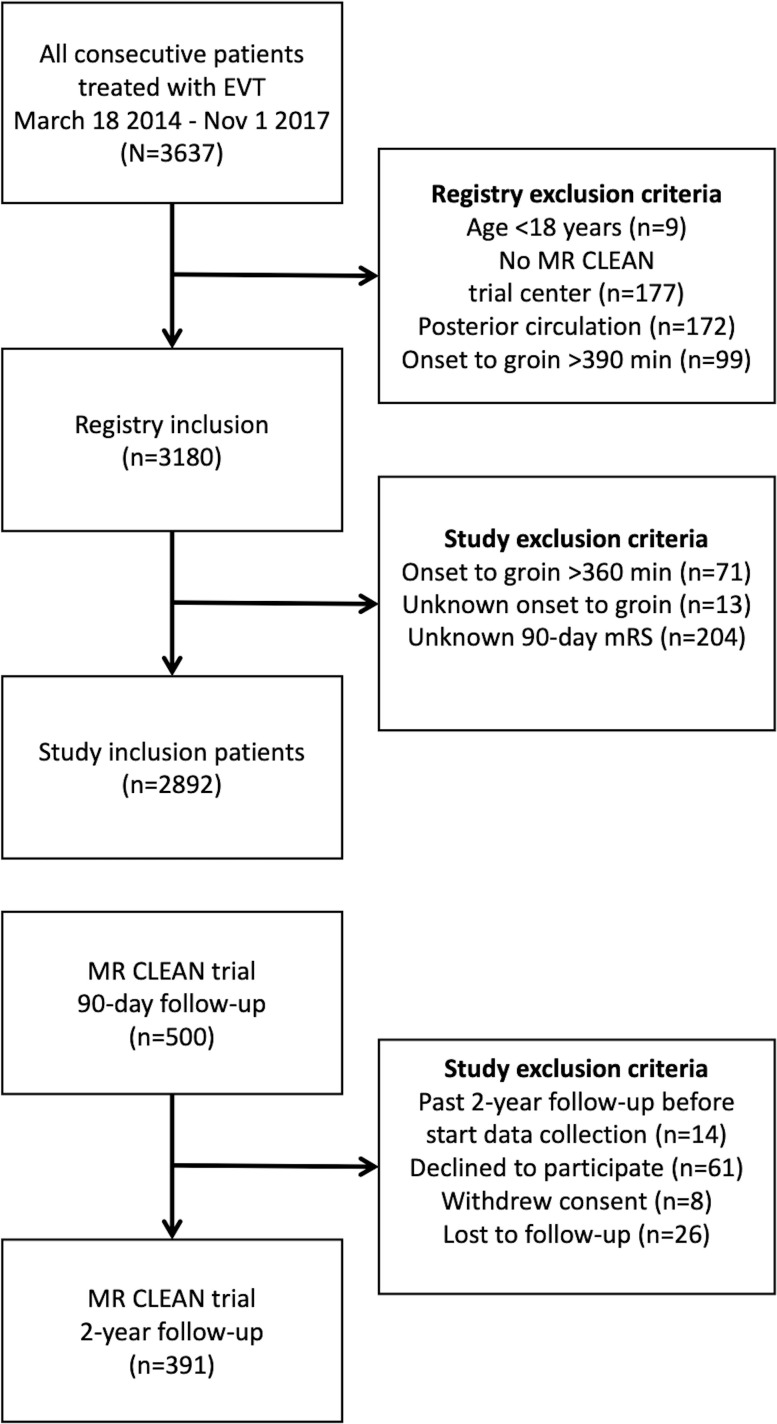
Data and inclusion. In this study data from the MR CLEAN Registry (part 1 and 2), MR CLEAN trial, and 2-year follow-up from the MR CLEAN trial were used. For this study, additional exclusion criteria were formulated for the MR CLEAN Registry data. EVT, endovascular treatment; mRS, modified Rankin Scale.

Input parameters of the Markov model were retrieved retrospectively from the MR CLEAN Registry part 1 and 2,^15^ and 2-year follow-up data from the MR CLEAN trial,^22 23^ public data,^24^ and literature.^25 26^ Patients from the MR CLEAN Registry were included based on the following criteria: LVO in the anterior circulation, treatment in MR CLEAN trial center, age ≥18 years, available 90-day mRS, available time from onset to groin puncture, and time from onset to groin puncture ≤360 min (figure 2). The data collection protocols have been published, and ethics committee and research board approval has previously been received in compliance with the Declaration of Helsinki.^15 27 28^

### Probabilities

The data of 2892 patients in the MR CLEAN Registry were used to retrieve the per-hour of time from onset to groin puncture and the probability of mRS sub-scores 90 days after EVT (figure 1A). Yearly mortality rates of the Dutch population by age and gender were retrieved from forecasts of the Royal Dutch Actuarial Society starting in 2021.^24^ Subsequently, published hazard rates (HR) of additional mortality related to mRS and years after index stroke were used to compute all-cause, including recurrent stroke-related, mortality rates.^25^ The probability of a recurrent stroke for each year after an index stroke was retrieved from a population study.^26^ To prevent double inclusion of stroke-related mortality in the model, death (mRS 6) was not modeled as an outcome after recurrent stroke. Instead, the HR by Hong et al on all-cause mortality was used, which included recurrent stroke-related mortality and other causes of death.^25^ Functional outcome at 90 days excluding death (mRS6) of the MR CLEAN trial control arm (n=267) was used to compute the mRS distribution after stroke recurrence (figure 1).^22^ As death could not occur and mRS could only remain equal or deteriorate, the outcomes of the MR CLEAN trial control arm were normalized for the available outcome options (online supplemental table S1).

10.1136/neurintsurg-2020-017017.supp1 Supplementary data

### Costs and QALYs

Two-year follow-up data of the MR CLEAN trial (n=391) was used to estimate QALYs and costs per unit of time in separate mRS sub-scores. Data was collected at 3, 6, 12, 18, and 24 months' post-index stroke and included utility scores computed from EuroQoL 5D and cost questionnaires. A QALY generally is a value between 0 and 1 where 0 implies death and 1 perfect health during 1 year. However, in our reference data it was also possible to have a negative QALY since some health situations were perceived by the patients as worse than death. Per patient, the average of each mRS/utility score combination across different time points was used as the QALY estimate. The online supplemental material contains more information on the data collection and computations with costs and utility scores previously collected by van den Berg et al.^27^ In short, costs were computed from a healthcare payer perspective and included: acute setting treatment cost, in-hospital costs, outpatient clinic visits, rehabilitation, formal homecare, and long-term institutionalized costs. The mRS at 90 days and 18 months were used as reference points for the calculation of costs per mRS-related health state for the first and second year after index AIS, respectively. Follow-up costs per mRS from the third year onward were assumed equal to costs of the second year excluding rehabilitation costs (online supplemental table S1). Mean costs and QALY values were used to perform baseline simulations. Historical inflation rates from the Dutch Central Bureau of Statistics were used to adjust the reported costs with reference year 2015 to the reference simulation start year of this study (2021).^27^

### Sensitivity analyses

One-way sensitivity analyses were performed to assess the degree of change in outcome due to change of a single input parameter. Outcome measures were reported for an increase and decrease of 10% with respect to the baseline input parameters. For age of the simulated population, a deviation of 4 years from the baseline age was used. Deviations in age were only used in the long-term model, but the effect of age on 90-day mRS was not included in this study. A probabilistic sensitivity analysis was performed to assess input parameter uncertainty. Online supplemental table S1 includes the distribution type per input parameter. 10 000 s order Monte Carlo simulations were used to estimate the certainty of the estimates. External validation with respect to simulated mRS and reported mRS in contemporary follow-up data of endovascular treated patients was performed.^23 29 30^ In the external validation the proportion of patients with good functional outcome (mRS ≤2), poor functional outcome but not deceased (mRS 3–5), or death (mRS 6) were compared with follow-up results from REVASCAT (1 year),^29^ MR CLEAN (2 years),^23^ and a study by Clua-Espuny et al (5 years).^30^

### Population effects

An estimate was made to represent the effects of 1 min of faster EVT for the Dutch population based on the PSA results. For this estimate the total population in the MR CLEAN registry part 1 and 2 (n=3279) included in 43 months was used to compute the yearly number of LVO patients that receive EVT (n=887) and thus could benefit from faster EVT. The outcome on the population level was computed by taking a weighted average based on the prevalence of each hour delay to groin puncture in the MR CLEAN registry.

### Outcome measures

The primary outcome measures used were Net Monetary Value (NMV), a single aggregated value for costs, and QALYs where QALYs are converted to a monetary value NMV=QALYs*willingness to pay - costs, per hour delay from onset to groin puncture, and Net Monetary Benefit (NMB, the NMV difference between each hour): at a willingness to pay threshold of €80 000 per QALY.^20^ Secondary outcome measures were QALYs and costs. Results were presented as cumulative values over the 5-year simulated period. Outcome measures were computed per hour delay of time from onset to groin puncture and reported for 1 min, 10 min, and 1 hour of faster EVT. Differences in outcome measures between the hours were used to compute outcome measures of faster EVT. Transforming outcomes per hour of faster EVT to per minute includes an assumption of constant differences between hours. Per minute of faster EVT, additional days of disability-free life (additional days in perfect health; QALYs/365), change in costs, and NMB were computed. PSA results were reported as median with IQR. For descriptive statistics (online supplemental table S2) distributions across the 6-hour delay were statistically compared with ANOVA, Kruskall–Wallis, and Chi-squared tests for normal distributed continuous, non-normal distributed continuous, and categorical distributed baseline variables, respectively.

### Model input parameters

Online supplemental table S1 in the online supplementary material depicts all parameters used for baseline simulations and probabilistic sensitivity analyses.

## Results

### Descriptive statistics

Online supplemental table S2 contains descriptive statistics of 2892 MR CLEAN registry patients per hour of delay from onset to groin puncture. Age, clinical (NIHSS), and radiological (ASPECTS) parameters were significantly associated with delay to EVT. The proportion of directly referred patients was significantly lower in the 4th–6th hours of delay from onset to groin puncture. Furthermore, intravenous thrombolysis was administered to 77.4%, and 61.3% of the entire population were directly referred to an EVT-capable center.

### Baseline simulations and one-way sensitivity analyses

Costs per hour of delay remained roughly equal, while QALYs and thus NMV decreased (online supplemental table S3). Each minute of faster EVT resulted in an NMB of €242 (per hour: €14,519). This was mainly due to a 1.4 gain of disability free life-days (0.224 QALYs per hour). Costs changed with -€41 per minute of faster EVT (per hour: -€2,433).

The one-way sensitivity analysis revealed that age at the start of simulations, QALYs per year in mRS 0–4, and costs per year in mRS 3–4 affect NMB most. Costs in the first year in mRS 2–4 affected NMB more than costs made in the subsequent 4 years, except for costs in mRS 4. Online supplemental figure S1 in the online supplementary material depicts the one-way sensitivity analyses results.

### Probabilistic sensitivity analysis

**Figure 3:**
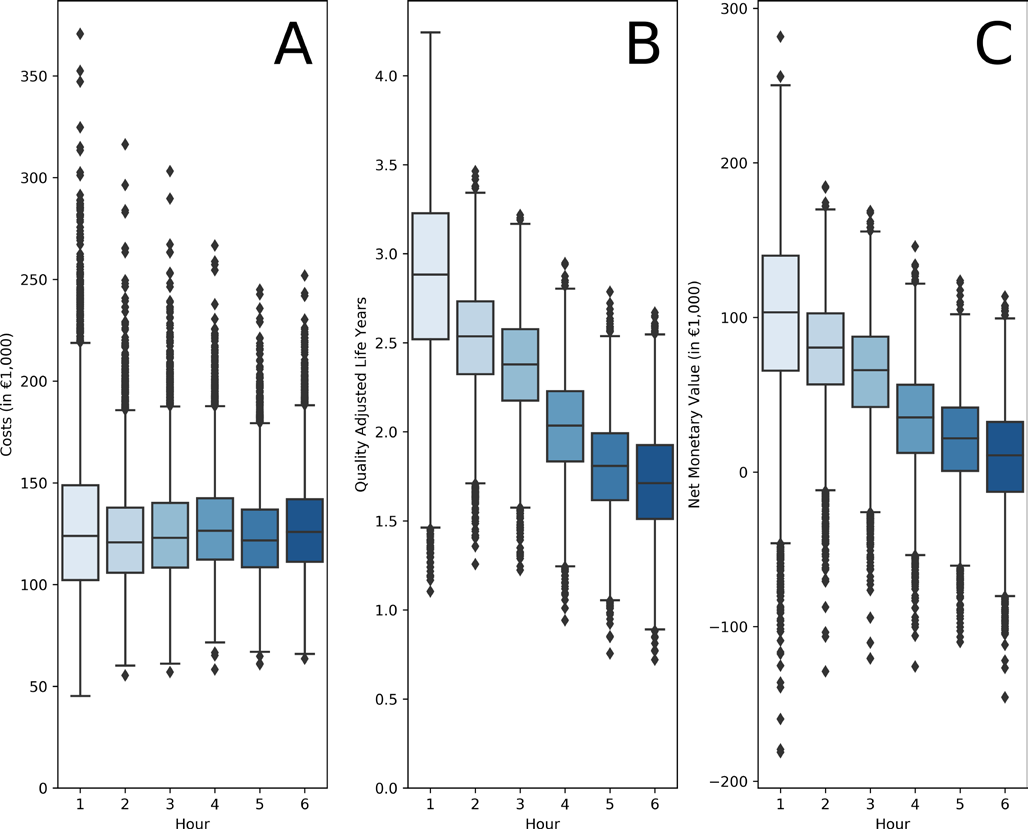
Probabilistic sensitivity analysis results per hour of delay from onset to groin puncture: Costs (A), quality-adjusted life-year (QALY) (B), and Net Monetary Value (C) per hour time from onset to groin puncture.

Similar to the baseline simulation results, costs remained stable while QALYs and thus NMV decreased per hour of delay in the PSA (figure 3). Faster EVT resulted in no change in costs (per minute median: -€15, IQR: -€65;€33 – per hour median: -€969, IQR: -€3,897;€1,972), a gain in health (per minute disability-free life-days gained median: 1.3, IQR: 1.0;1.6 – per hour QALYs gained median: 0.22, IQR: 0.17;0.27), resulting in a positive NMB (per minute median: €309, IQR: €226;389 – per hour median: €18 513 IQR: €13,574;€23,376). Results of faster EVT for differences between separate hours of time to groin and the outcome for the Dutch population are depicted in table 1. Faster EVT between the third and fourth hour had a more profound effect on disability-free life-days and NMB. For an expected number of 887 EVT-eligible AIS patients in the Dutch population yearly, delivery of EVT by 1 min faster would result in a median QALY gain of 3.5 (IQR: 2.1;4.9 – per hour median: 210.1, IQR: 124.0;294.8), and a per minute median NMB of €287 324 (IQR: €146,270;€428,547 – per hour median: €17,239,435, IQR: €8,776,181;€25,712,803).

**Table 1:** Probabilistic sensitivity analysis results per hour of faster treatment

Hour difference	Costs in € median (IQR)	QALY gained median (IQR)	NMB in € median (IQR)
First – second hour	2633 (−10,957;18,613)	0.36 (0.09;0.61)	23 799 (−3004;49,133)
Second – third hour	−2219 (−6,651;2,240)	0.15 (0.07;0.23)	14 052 (6,809;21,714)
Third – fourth hour	−3228 (−7,274;1,026)	0.34 (0.28;0.40)	30 143 (23,732;36,679)
Fourth – fifth hour	4666 (440;9,078)	0.22 (0.16;0.29)	13 125 (6,353;19,682)
Fifth – sixth hour	−3860 (−10,108;2,261)	0.09 (−0.01;0.18)	10 560 (1,708;19,971)
Per hour faster treatment*	−969 (−3,897;1,972)	0.22 (0.17;0.27)	18 513 (13,574;23,376)
Per 10 min faster treatment*	−151 (−649; 329)	13.5 (10.5;16.4)†	3085 (2,262;3,896)
Per minute faster treatment*	−15 (−65;33)	1.3 (1.0;1.6)†	309 (226;389)
Population outcome per hour of earlier treatment‡	−865,387 (−5,848,900;4,459,552)	210.1 (124.0;294.8)	17 239 435 (8,776,181;25,712,803)
Population outcome per 10 min of earlier treatment‡	−144,231 (−974,817;743,258)	35.0 (20.7;49.1)	2 873 239 (1,462,696;4,285,467)
Population outcome per minute of earlier treatment§	−14,423 (−97,482;74,326)	3.5 (2.1;4.9)	287 324 (146,270;428,547)

*For each simulation the median value of the five differences between 6 hours was taken.

†The per minute QALY results are depicted as disability-free life days gained (DALY). This is the daily value of a QALY (DALY=QALY/365).

‡Outcome for the Dutch LVO stroke population if faster EVT is performed.

### External validation of simulated MRS distributions

In online supplemental figure S2 in the online supplementary material the external validation results are depicted. Good functional outcome (mRS ≤2; online supplemental figure S2A) and mortality (mRS 6; online supplemental figure S2C) at 1 and 2 years were comparable with results from the REVASCAT and MR CLEAN follow-up studies.^23 29^ Observed values were similar to simulated values of 3-hour delay to groin puncture. Mortality (mRS 6; online supplemental figure S2C) at 5= year follow-up was higher in the simulated data compared with the study by Clua-Espuny et al.^30^

## Discussion

Delay of time from onset to groin puncture results in a loss of health (QALYs) that accumulates over the years following an acute LVO stroke setting. Faster EVT will result in a gain in health, while costs from a healthcare payer perspective remain stable. Thus, faster EVT is cost effective. We have indicated that an NMB of €309 per minute faster EVT may be achieved, which, extrapolated to the Dutch population, equals an annual NMB of €287 324. Since the treatment effect of EVT in the Dutch MR CLEAN trial and Registry was lower than that of its international peers, it seems likely that the NMB per minute faster EVT is even higher in populations with higher treatment effect of EVT.

Even though only the potential benefits and not the costs of realizing faster EVT were considered, this study provides a strong argument to invest more in stroke logistics. Depending on the approach to realize faster EVT it seems likely that additional costs will be made. For example, better ambulance coverage or public awareness of stroke symptoms might be used for faster EVT delivery, which requires investments in ambulance coverage and educational programs that are not included in this study. Furthermore, it should be considered that in per euro invested in expediting EVT there might be diminishing returns with respect to the realized minutes of faster EVT.

The paradox of higher lifetime healthcare costs due to longer survival caused no cost savings as a result of faster EVT in this study. Patients with faster EVT had an improved functional outcome (mRS) and life expectancy, however, additional healthcare costs were made in the long term. Faster EVT in the population treated between the third and fourth hour of onset to groin puncture was related to a larger health effect than that of differences between other hours. This could be related to the proportion of directly referred patients to an EVT-capable stroke center (online supplemental table S2): a potential selection of indirectly referred patients could have occurred. Although a clear relationship between faster EVT and NMB was found, this result was very sensitive to age. An increase in age resulted in an exponential decrease of the cost effectiveness of faster EVT in terms of NMB, which is in line with the findings by Wu et al where the cost effectiveness of reperfusion after EVT decreased with an increase in age.^31^ In addition, the effect of age in this study is likely to be an underestimation since the modeled effect of age only considered mRS changes in the long run (after 90 days' post-stroke), whereas it is known that age is also related to poor functional outcome at 90 days.^4^

Findings from this study are in accordance with the US-based population study by Kunz et al,^14^ and the indirect effect of time on the cost-effectiveness analyses for M2 occlusions by Khunte et al.^32^ The exact quantitative value of faster EVT was, however, much less (NMB median: $1,059; 95% prediction interval: $555-$1,485) than the study by Kunz et al. This difference in NMB per minute of faster EVT was mainly due to differences in follow-up simulation time and cost computations: Kunz et al used life-time simulations and included both societal and healthcare costs, while 5-year follow-up and only direct healthcare costs were used in this study. This timespan was chosen to retrieve a more conservative and certain estimation of the value of time, since the simulation of longer follow-up results in the accumulation of probability estimate errors. Kunz et al also found that age was the major factor affecting NMB of faster treatment.^14^ The comparison of simulated results with observed long-term mRS distributions revealed a comparable proportion of good functional outcome but a higher mortality rate in the baseline simulations than observed rates in the validation studies considered.^23 29 30^ Although this might implicate an overestimated mortality rate, a lower age in two of three validation studies (age in Clua-Espuny et al^30^; MR CLEAN; REVASCAT: 69.5;65.5;67.2) could explain the lower mortality rate in these studies. In addition, these validation studies either were retrospective in design and thus prone to selection bias (Clua-Espuny et al^30^), had a large loss to follow-up (MR CLEAN, 16.7%), or had a relatively low sample size (REVASCAT, n=103).

The lack of data regarding the long-term decline of mRS, stroke recurrence, and mortality in patients with LVO undergoing EVT remains a shortcoming of this study. If survival and time in different mRS states differ in the long-run costs and health effects also alter significantly. Another limitation of this study was the lack of recent data on healthcare spending after a 2-year follow-up period and societal costs. To improve the accuracy of cost-effectiveness studies in stroke, future research should aim at retrieving better cost estimates from a healthcare payer and societal perspective related to mRS during multiple years after an initial stroke. However, if the relative cost differences related to the mRS Markov states remain similar, it is in the line of expectation that the results of this study would not differ. Furthermore, mRS decline and mortality related to mRS in time should be studied more extensively.

The results of this study should be seen as the potential benefit of faster EVT at a population level: 1 min of faster EVT does not necessarily benefit every patient equally. Since age has a large effect on the cost effectiveness of faster treatment, the extrapolation of findings from this study to other, potentially much older, populations should be performed with caution. Furthermore, the high percentage of direct referral to an EVT-capable center and intravenous thrombolysis administration were not included in this study. Deviations of those percentages in other populations might result in a different EVT treatment effect/time relationship which, in turn, affects cost effectiveness and clinical outcomes. However, randomized controlled trial-based cost effectiveness analyses are required to determine the relationship between direct referral to EVT-capable centers, thrombolysis administration, and faster EVT. Nevertheless, we believe that with the available (public) information this study gives a fine-grained representation of mRS and a conservative cost and health effect estimate that stresses the necessity for healthcare payers to extensively invest in faster EVT delivery.

## Conclusion

Saving time to groin puncture has the potential to improve health while healthcare costs may be expected to remain stable over 5-year follow-up. At a willingness to pay of €80 000 per QALY, 1 min of faster treatment is equivalent to 1.3 disability-free life-days saved and a NMB of €309 in a median 69-year-old Dutch population.

10.1136/neurintsurg-2020-017017.supp2 Supplementary data

## Data Availability

Data are available upon reasonable request. Data used in this study can be accessed upon request, more information is available at https://www.mrclean-trial.org/home.html.
