# Supplementary material for: Quantified health and cost effects of faster endovascular treatment for large vessel ischemic stroke patients in the Netherlands
Source: J Neurointerv Surg. 2021 Jan 21;13(12):1099–105. doi: 10.1136/neurintsurg-2020-017017 (PMC8606465; doi:10.1136/neurintsurg-2020-017017)
Supplement: Supplementary data [file neurintsurg-2020-017017supp001.pdf]

Online supplementary material

Cost and QALY computation

This appendix contains information on the data collection and computations with costs and utility scores previously collected by van den Berg et al. currently under review.

Costs per unit of care: Per unit of care the costs were extracted from the Dutch costing manual for health care research 2015, hospital registration system, and data published by the institute of Medical Technological Assessment (iMTA) Rotterdam the Netherlands. These per unit costs were used to compute the total costs for the reference year 2015.

Acute setting units of care used: In the acute stroke setting the following units of care were included to compute acute setting costs. Units of care related to the intervention were deterministically defined. The following intervention care was included: 1 (neuro-) interventionist, 1 anesthesiologist, 2 radiology assistants, 2 anesthesia assistants during 1.5 hours of treatment delivery. 1 CTA scan was added, thrombectomy materials (guide wire, balloon, stent retriever or aspiration device), angiographic materials, vascular closure devices, and heparin. Additional costs for general anesthesia were added; we thus assumed all procedure s were conducted with general anesthesia (only marginal cost difference). The total acute treatment costs, thrombolysis excluded, were inflated by 42% according to represent overhead costs, the percentage was derived from iMTA (maximum overhead percentage academical hospitals). Finally, the costs of thrombolysis delivery were extracted from Medicijnkosten.nl 2016.

Long term setting units of care used: In-hospital care use, outpatient clinic visits, rehabilitation, formal homecare, and long-term institutionalized care were extracted from patient questionnaires and report forms, medical records, and the hospital information systems.

Quality-adjusted life year (QALY): EuroQol5D questionnaires were used to compute utility scores between -0.329 and 1 at 3, 6, 9, 12, 18, and 24 months. The negative utility refers to a situation that is worse than death (QALY=0), a QALY of 1 refers to perfect health. Per patient, the EQ5D utility was averaged across different time points per mRS group. The resulting value was used as the QALYs per unit of time in an mRS group.

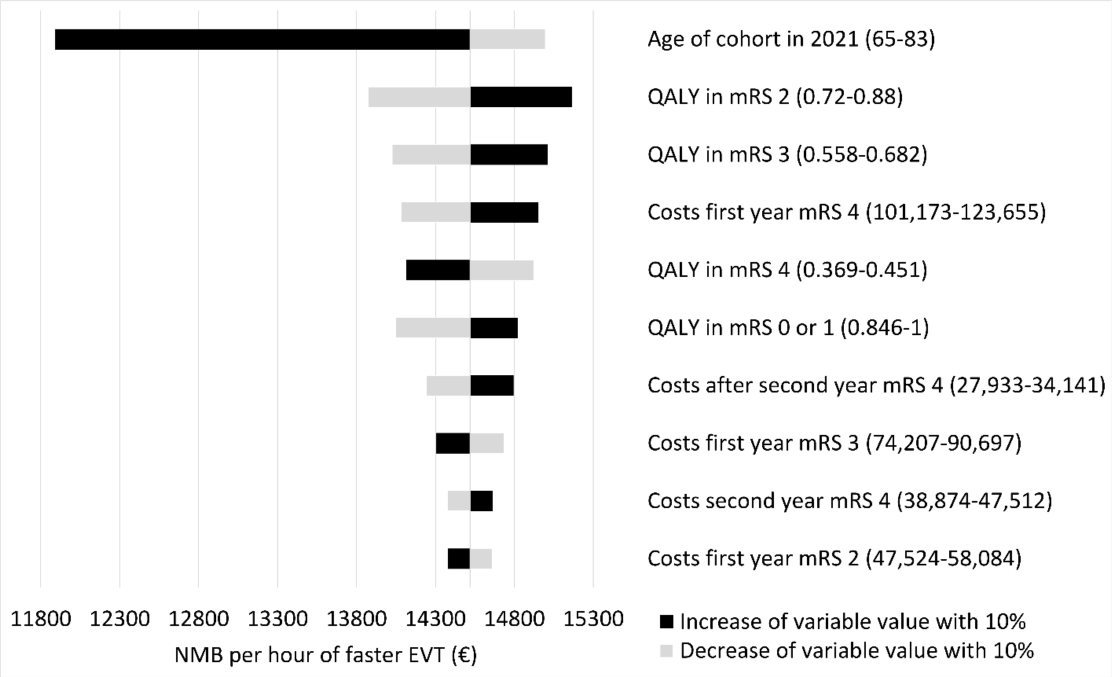

Figure S1. Net monetary benefit Tornado diagram: The effects in terms of NMB per hour of faster EVT due to a 10% increase (red) or decrease (blue) in model input parameters is depicted in a descending order for the 10 most impactful model parameters. The middle of the tornado diagram used the median NMB (€14,519) found in the baseline simulations. NMB: net monetary benefit, QALY: Quality-adjusted life years.

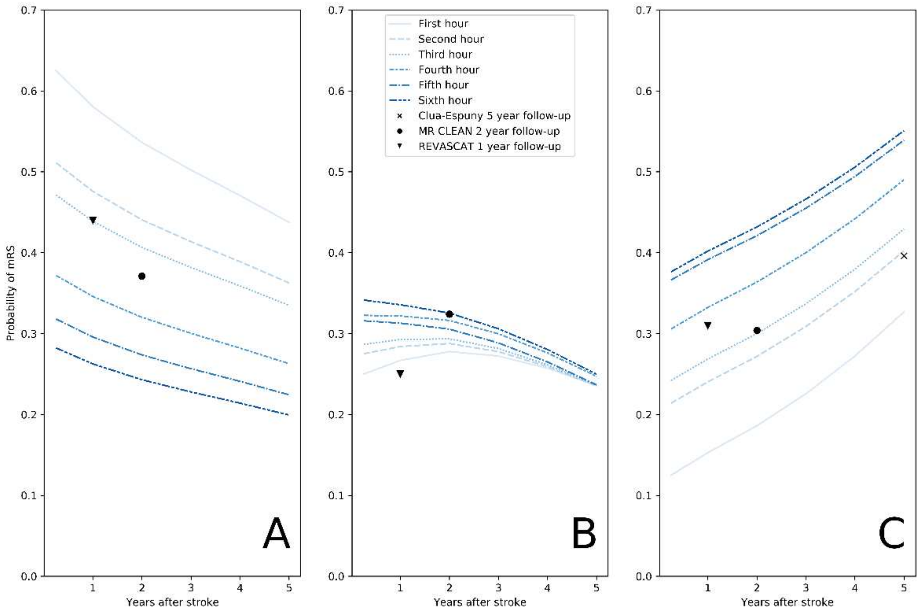

Figure S2. Baseline simulation external validation: Per hour of time from onset to groin puncture the proportion of simulated patients in mRS 0-2 (A), mRS 3-5 (B), and mRS 6 (C) was depicted. Published long term follow-up of EVT treated patients from Clua-Espuny et al. (cross), MR CLEAN (circle), and REVASCAT (triangle) were added. Due to a joint reporting of mRS 5-6 in the REVASCAT follow-up, pane B includes mR 3-4 and pane C mRS 5-6. mRS: modified Rankin Scale.

Variables	Baseline value	Distribution	Data source
	Outcome<br>number of patients (%)		
mRS after 0-60 minutes time to groin (mRS: 01/2/3/4/5/6)	4(25.0)/6(37.5)/2(12.5)/<br>2(12.5)/0(0)/2(12.5)	Dirichlet	MR CLEAN registry
mRS after 61-120 minutes time to groin (mRS: 01/2/3/4/5/6)	106(29.4)/78(21.7)/53(14.7)/<br>30(8.3)/16(4.4)/77(21.4)	Dirichlet	MR CLEAN registry
mRS after 121-180 minutes time to groin (mRS: 01/2/3/4/5/6)	263(27.9)/181(19.2)/116(12.3)/<br>112(11.9)/42(4.5)/228(24.2)	Dirichlet	MR CLEAN registry
mRS after 181-240 minutes time to groin (mRS: 01/2/3/4/5/6)	162(19.5)/147(17.7)/116(14.0)/<br>100(12.0)/52(6.3)/254(30.6)	Dirichlet	MR CLEAN registry
mRS after 241-300 minutes time to groin (mRS: 01/2/3/4/5/6)	73(16.0)/72(15.8)/64(14.0)/<br>49(10.7)/31(6.8)/167(36.6)	Dirichlet	MR CLEAN registry
mRS after 301-360 minutes time to groin (mRS: 01/2/3/4/5/6)	42(14.6)/39(13.6)/36(12.5)/<br>48(16.7)/14(4.9)/108(37.6)	Dirichlet	MR CLEAN registry
mRS no EVT (after recurrent stroke; by mRS: 01/2/3/4/5)	16(7.7)/35(16.8)/44(21.2)/<br>81(38.9)/32(15.4)	Dirichlet	MR CLEAN trial
IVT given (per hour 1/2/3/4/5/6) †	11(68.8)/265 (73.6)/735 (78.5)/663 (79.9)/352 (77.5)/206 (71.8)	Beta	MR CLEAN registry
Baseline recurrent stroke	Dependent on years after index ischemic stroke	Fixed values	Pennlert et al. supplementary material
HR recurrent stroke (by mRS: 01/2/3/4/5)	Age (OR 1.03 per year) dependent	Log-normal	Pennlert et al.
Baseline mortality	Age, gender, and year dependent	Fixed values	Dutch Royal Actuarian Society
HR mortality (by mRS: 01/2/3/4/5)	1.54/2.17/3.18/4.55/6.55	Log-normal	Hong et al.
Inflation rate in % per year (2015/2016/2017/2018/ 2019/2020 and after)	0.6/0.3/1.4/2.6/1.7	Fixed value	CBS
	Costs and QALY mean(std) ‡		
Costs EVT	9924.50	Fixed value	CLOT MR CLEAN
Costs IVT†	950.82	Fixed value	CLOT MR CLEAN

Costs year 1 (by mRS: 01/2/3/4/5/6)	33,402(31,930)/52,804(23,571)/ 82,452(35,333)/112,414(35,786)/ 96,640(30,463)/21,112(17,350)	Gamma	CLOT MR CLEAN
Costs year 2 (by mRS: 01/2/3/4/5/6)	5,934(15,918)/8,543(14,844)/ 19,235(15,999)/43,193(45,640)/ 56,425/24,252)/423(3,196)	Gamma	CLOT MR CLEAN
Costs year 2 onward (by mRS: 01/2/3/4/5/6) §	3,633(9,087)/7,318(13,770)/ 16,276(11,753)/31,037(19,928)/ 54,997(24,874)/374(3,118)	Gamma	CLOT MR CLEAN
QALY (by mRS: 01/2/3/4/5/6) ¶	0.94(0.09)/0.80(0.17)/0.68(0.24)/ 0.39(0.26)/0.24(0.25)/0(0.01)	Beta	CLOT MR CLEAN

Table S1. Model input parameters: †: Probability of IVT per hour of onset time to groin and costs of IVT were combined to compute additional treatment costs on top of EVT, patients referred from medical centers without EVT capabilities with >4.5 hours onset time to groin could also have received IVT. ‡:Mean and standard deviation (std) of cost and QALY estimates were used to construct gamma distributions and beta distributions respectively. §: Rehabilitation costs were close to zero after the second year follow up year and were assumed zero. Adding to this assumption is that rehabilitation is not seemed effective more than 2 years after index stroke. ¶: mRS 6 - QALY association depicts the association for the year of death, all subsequent years QALY was assumed zero. CBS: Dutch Central Bureau of Statistics.

	Hours of delay from onset to groin puncture							p-value
	**All hours**	**First**	**Second**	**Third**	**Fourth**	**Fifth**	**Sixth**	
Total count (n)	2892	16	360	942	831	456	287	
Age (years)	70 (14.0)	63 (15.0)	69 (14.1)	68 (14.2)	71 (13.1)	71 (14.2)	71 (15.1)	<1e-4
NIHSS	16 (11-20)	10 (5-15)	16 (11-20)	16 (12-20)	16 (11-19)	15 (11-19)	16 (10-20)	0.03
ASPECTS	9 (8-10)	10 (9-10)	9 (8-10)	9 (8-10)	9 (7-10)	9 (7-10)	9 (7-10)	<1e-4
Sex female	1373 (47.5%)	8 (50.0%)	145 (40.3%)	427 (45.3%)	403 (48.5%)	227 (49.8%)	163 (56.8%)	<1e-3
Collateral score								0.63
0	168 (6.2%)	0 (0.0%)	19 (5.5%)	56 (6.4%)	46 (5.9%)	32 (7.5%)	15 (5.7%)	
1	987 (36.3%)	4 (30.8%)	118 (34.0%)	314 (35.7%)	301 (38.7%)	166 (38.7%)	84 (32.1%)	
2	1038 (38.3%)	8 (61.5%)	140 (40.3%)	340 (38.7%)	292 (37.5%)	152 (35.4%)	106 (40.5%)	
3	516 (19.0%)	1 (7.7%)	70 (20.2%)	169 (19.2%)	139 (17.9%)	79 (18.4%)	57 (21.8%)	
mRS prior to stroke								0.04
0	2563 (73.6%)	12 (75.0%)	247 (69.2%)	655 (70.7%)	547 (67.4%)	274 (61.6%)	173 (63.6%)	
1	377 (10.8%)	3 (18.8%)	38 (10.6%)	126 (13.6%)	106 (13.1%)	59 (13.3%)	45 (16.5%)	
2	213 (6.1%)	1 (6.2%)	32 (9.0%)	57 (6.1%)	68 (8.4%)	38 (8.5%)	17 (6.2%)	
3	188 (5.4%)	0 (0.0%)	30 (8.4%)	50 (5.4%)	47 (5.8%)	39 (8.8%)	22 (8.1%)	
4	116 (3.3%)	0 (0.0%)	7 (2.0%)	32 (3.5%)	35 (4.3%)	30 (6.7%)	12 (4.4%)	
5	27 (0.8%)	0 (0.0%)	3 (0.8%)	7 (0.8%)	9 (1.1%)	5 (1.1%)	3 (1.1%)	
Infarct in left hemisphere	1277 (47.0%)	3 (18.8%)	167 (49.1%)	405 (45.7%)	383 (48.7%)	191 (44.8%)	128 (48.5%)	0.14
IVT	2232 (77.4%)	11 (68.8%)	265 (73.6%)	735 (78.5%)	663 (79.9%)	352 (77.5%)	206 (71.8%)	0.03
Off-hours	1827 (63.2%)	3 (18.8%)	178 (49.4%)	574 (60.9%)	543 (65.3%)	325 (71.3%)	204 (71.1%)	<1e-5
Directly referred to EVT capable center	1773 (61.3%)	14 (87.5%)	344 (95.6%)	616 (65.4%)	416 (50.1%)	226 (49.6%)	157 (54.7%)	<1e-5
Time from onset to IVT	98.7 (53.7)	20 (10.9)	58 (17.0)	72 (24.7)	98 (35.7)	138 (54.2)	188 (65.2)	<1e-5

Time from onset to groin puncture	199 (69.3)	41 (14.1)	102 (14.7)	153 (17.0)	210 (16.8)	269 (17.2)	330 (17.5)	<1e-5
Time from onset to reperfusion	254 (75.7)	96 (31.4)	160 (38.7)	210 (38.4)	264 (39.3)	324 (34.1)	383 (37.2)	<1e-5

Table S2. Descriptive statistics per hour of delay from onset to groin puncture. Descriptive statistics are either presented as: mean (std), median (25th percentile-75th percentile), count (percentage%). EVT: endovascular treatment, IVT: intravenous treatment, NIHSS: NIH stroke scale, ASPECTS: Alberta stroke program early CT score, ER: emergency room, off-hours implied if the patient was admitted between 17:00-08:00 on week-days, in the weekend, or on holidays.

Hours from onset to groin puncture	Costs (€)	QALY	NMV
First (≤60 minutes)	125,254	2.848	102,564
Second (61-120 minutes)	120,700	2.509	79,993
Third (121-180 minutes)	123,133	2.358	65,474
Fourth (181-240 minutes)	126,118	2.015	35,081
Fifth (241-300 minutes)	121,631	1.791	21,614
Sixth (301-360 minutes)	125,853	1.703	10,395

Table S3. Baseline simulation results per hour of delay from onset to groin puncture: QALY: Quality-adjusted life year, NMV: Net Monetary Value.
