# Supplementary material for: Quantified health and cost effects of faster endovascular treatment for large vessel ischemic stroke patients in the Netherlands
Source: J Neurointerv Surg. 2021 Jan 21;13(12):1099–105. doi: 10.1136/neurintsurg-2020-017017 (PMC8606465; doi:10.1136/neurintsurg-2020-017017)
Supplement: Supplementary data [file neurintsurg-2020-017017supp002.pdf]

Section/item	Item No	Recommendation	Reported on page No(p)/ line No(l)
**Title and abstract**			
Title	1	Identify the study as an economic valuation or use more specific terms such as "cost-effectiveness analysis", and describe the interventions compared.	P1 l3
Abstract	2	Provide a structured summary of objectives, perspective, setting, methods (including study design and inputs), results (including base case and uncertainty analyses), and conclusions.	P1l30 – p2 l11
**Introduction**			
Background and objectives	3	Provide an explicit statement of the broader context for the study. Present the study question and its relevance for health policy or practice decisions.	P2 l30 – p3 l27
**Methods**			
Target population and subgroups	4	Describe characteristics of the base case population and subgroups analysed, including why they were chosen.	P3 l34-44
Setting and location	5	State relevant aspects of the system(s) in which the decision(s) need(s) to be made.	P3 l13–24, p1 l1, p4 l12-35
Study perspective	6	Describe the perspective of the study and relate this to the costs being evaluated.	P5 l53 – p6 l17
Comparators	7	Describe the interventions or strategies being compared and state why they were chosen.	P5 l5-8
Time horizon	8	State the time horizon(s) over which costs and consequences are being evaluated and say why appropriate.	P4 |12-21
Discount rate	9	Report the choice of discount rate(s) used for costs and outcomes and say why appropriate.	P4 l24-30
Choice of health outcomes	10	Describe what outcomes were used as the measure(s) of benefit in the evaluation and their relevance for the type of analysis performed.	p7 l21-53
Measurement of effectiveness	11a	*Single study-based estimates:* Describe fully the design features of the single effectiveness study and why the single study was a sufficient source of clinical effectiveness data.	Not applicable
	11b	*Synthesis-based estimates:* Describe fully the methods used for identification of included studies and synthesis of clinical effectiveness data.	P4 l39-52
Measurement and valuation of preference based outcomes	12	If applicable, describe the population and methods used to elicit preferences for outcomes.	Not applicable

Estimating resources and costs	13a	*Single study-based economic evaluation:* Describe approaches used to estimate resource use associated with the alternative interventions. Describe primary or secondary research methods for valuing each resource item in terms of its unit cost. Describe any adjustments made to approximate to opportunity costs.	Not applicable
	13b	*Model-based economic evaluation:* Describe approaches and data sources used to estimate resource use associated with model health states. Describe primary or secondary research methods for valuing each resource item in terms of its unit cost. Describe any adjustments made to approximate to opportunity costs.	P4 l40-55, p5 l6 – p6 l24, online supplement
Currency, price date, and conversion	14	Report the dates of the estimated resource quantities and unit costs. Describe methods for adjusting estimated unit costs to the year of reported costs if necessary. Describe methods for converting costs into a common currency base and the exchange rate.	P6 l 17-23
Choice of model	15	Describe and give reasons for the specific type of decision analytical model used. Providing a figure to show model structure is strongly recommended.	P3 l 47 – p4 l21, figure 1
Assumptions	16	Describe all structural or other assumptions underpinning the decision-analytical model.	P5|41 – p6|23
Analytical methods	17	Describe all analytical methods supporting the evaluation. This could include methods for dealing with skewed, missing, or censored data; extrapolation methods; methods for pooling data; approaches to validate or make adjustments (such as half cycle corrections) to a model; and methods for handling population heterogeneity and uncertainty.	P6 l 26 – p7|17
**Results**			
Study parameters	18	Report the values, ranges, references, and, if used, probability distributions for all parameters. Report reasons or sources for distributions used to represent uncertainty where appropriate. Providing a table to show the input values is strongly recommended.	Tables S1, S2 (supplement)
Incremental costs and outcomes	19	Report the values, ranges, references, and, if used, probability distributions for all parameters. Report reasons or sources for distributions used to represent uncertainty where appropriate. Providing a table to show the input values is strongly recommended.	Table 1, figures S1, S2 (supplement)

Characterising uncertainty	20a	*Single study-based economic evaluation:* Describe the effects of sampling uncertainty for the estimated incremental cost and incremental effectiveness parameters, together with the impact of methodological assumptions (such as discount rate, study perspective).	Not applicable
	20b	*Model-based economic evaluation:* Describe the effects on the results of uncertainty for all input parameters, and uncertainty related to the structure of the model and assumptions.	P8 l44 – p9|28, figure 3, table 1
Characterising heterogeneity	21	If applicable, report differences in costs, outcomes, or costeffectiveness that can be explained by variations between subgroups of patients with different baseline characteristics or other observed variability in effects that are not reducible by more information.	Not applicable
**Discussion**			
Study findings, limitations, generalisability, and current knowledge	22	Summarise key study findings and describe how they support the conclusions reached. Discuss limitations and the generalisability of the findings and how the findings fit with current knowledge.	P10|6-20
**Other**			
Source of funding	23	Describe how the study was funded and the role of the funder in the identification, design, conduct, and reporting of the analysis. Describe other non-monetary sources of support.	Acknowledgements and added disclosure
Conflicts of interest	24	Describe any potential for conflict of interest of study contributors in accordance with journal policy. In the absence of a journal policy, we recommend authors comply with International Committee of Medical Journal Editors recommendations.	Acknowledgements and added disclosure
